# Perineal fibroadenoma masquerading as a soft-tissue sarcoma: a case report

**DOI:** 10.1093/jscr/rjad358

**Published:** 2023-07-07

**Authors:** Amy Hannigan, Gabriella Wende, Ryan Cohen, Stephanie Chetrit

**Affiliations:** Department of General Surgery, St John of God Healthcare, Perth, Australia; King Edward Memorial Hospital, Perth, Australia; Department of General Surgery, St John of God Healthcare, Perth, Australia; School of Medicine, The University of Notre Dame, Fremantle, Australia; School of Biomedical Sciences, The University of Western, Perth, Australia; Department of General Surgery, St John of God Healthcare, Perth, Australia; School of Medicine, The University of Notre Dame, Fremantle, Australia

**Keywords:** Perineal fibroadenoma, Ectopic breast tissue, Soft tissue sarcoma

## Abstract

We report a case of a perineal fibroadenoma initially diagnosed on ultrasound and magnetic resonance imaging as a soft tissue sarcoma in a 35-year-old female. Following wide local excision, histopathology revealed the lesion as a vulval fibroadenoma. We provide an overview of the literature and highlight the need to consider fibroadenoma, arising from ectopic breast tissue, as an important differential for general surgeons and gynaecologists caring for patients with perineal masses.

## INTRODUCTION

Vulval fibroadenomas are rare benign tumours. We describe a case of perineal fibroadenoma masquerading as soft tissue sarcoma in a 35-year-old female.

## CASE REPORT

A 35-year-old female Gravida 0, Para 0, presented with a 2-year history of progressively enlarging painless perineal lump. There was a mobile, palpable, soft, non-fluctuant, non-tender mass located in the right perineum on examination.

**Figure 1:**
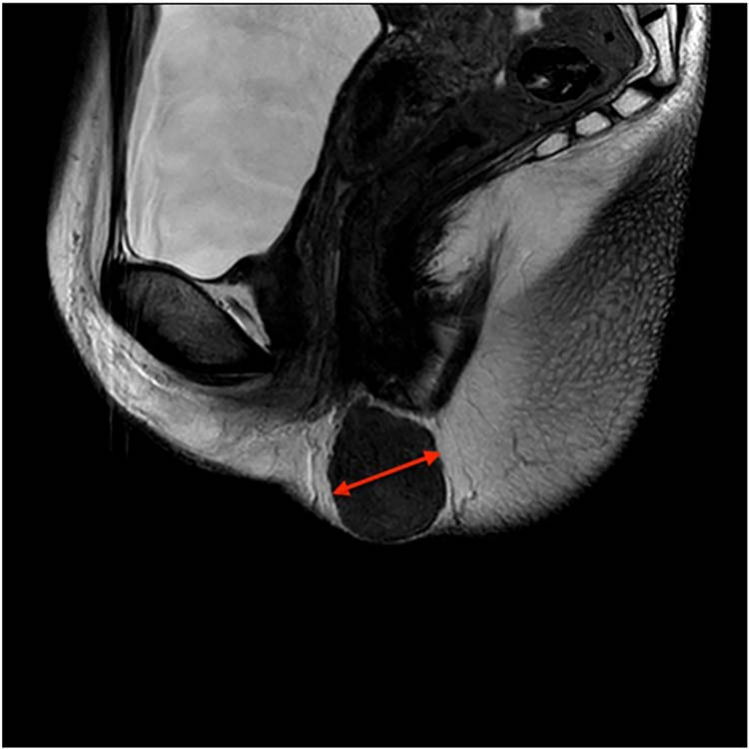
Coronal view MRI image of the lesion marked with a red arrow.

**Figure 2:**
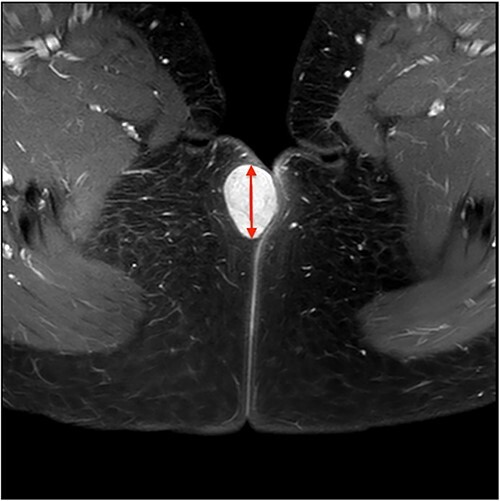
Axial view MRI image of the lesion marked with a red arrow.

On ultrasound, the lesion was described as a heterogeneous mass with complex internal vascularity, highly suggestive of malignancy. Further workup with magnetic resonance imaging (MRI) demonstrated a 36 × 28 × 23 mm mass at the posterior aspect of the right vulva visible on the coronal (Fig. 1) and axial view (Fig. 2). The lesion was abutting the skin surface but separate from the anus, vagina and urethra, and suspicious for soft-tissue sarcoma.

The patient was advised to undergo punch biopsy to further differentiate the lesion. However, she declined and instead opted for wide local excision, which proceeded without complication. Histopathology revealed the lesion to be a benign, mammary-type fibroadenoma arising from ectopic breast tissue. The specimen had clear margins with an intact fibrous pseudocapsule and contained no malignant cells.

## DISCUSSION

Fibroadenomas are the most common breast tumours in women, affecting up to 15% of women at some time in their lives, with the highest incidence between 20 and 30 years [1]. Several extramammary locations of fibroadenomas have been described in the literature (i.e. eyelid, nose, gallbladder and prostate) [2–5]; however, fibroadenomas in the vulval region are rare and are therefore at risk of being misdiagnosed and mismanaged.

The formation of ectopic breast tissue in any location other than along the mammary ridge is supported by two beliefs. One is that it represents a migratory arrest of breast primordium during chest wall development [6]; the other more commonly adopted belief introduced by Van der Putte [7] is that they develop from anogenital ‘sweat’ glands, of which histological appearance mimics those of mammary glands.

The significance of this theory is that, just like breast tissue, these anogenital glands have the potential to evolve into benign lesions like fibroadenomas or, in some cases, neoplastic lesions such as invasive carcinoma [8]. They, therefore, present a diagnostic and therapeutic dilemma for both general surgeons and gynaecologists.

This case was initially suspicious for sarcoma based on the clinical picture and MRI findings. Soft tissue sarcomas are rare tumours defined histologically as heterogeneous neoplasms arising from mesenchymal tissues. Perineal sarcomas represented 4.7% of all soft tissue sarcomas [9]. Clinically, these tumours are often painless swellings that progressively increase in size and can, therefore, mimic our case’s presentation.

Pre-operative diagnosis is particularly challenging because of the wide variety of tissues in the pelvic and perineal areas. A recent publication suggests a low-grade sarcoma should be a differential diagnosis for any perineal mass [10]. This author also reported the benefit of MRI with intravenous contrast as an imaging modality for the characterization and diagnosis of perineal soft-tissue lesions and assessing the position, depth and anatomical relationships of the lesion. On MRI, fibroadenomas typically appear as smooth masses with high signal intensity on T2-weighted images. However, fibroadenomas may have a contrast-enhancement pattern suggestive of malignancy in up to one-third of cases [11]. Definitive diagnosis often requires excisional biopsy or image-guided biopsy. On histopathology, fibroadenomas are comprised of glandular epithelium and specialized interlobular stroma of the terminal ductal lobular unit.

While the current gold standard treatment of symptomatic or rapidly enlarging breast fibroadenomas is surgical excision, there are no guidelines for the management of fibroadenomas of the perineum or anogenital region. The challenge of surgical resection is to obtain negative margins without causing disturbance to urinary or anorectal sphincter function.

Perineal fibroadenomas present a unique set of challenges, both in diagnosis and management. Their proximity to genitourinary and anorectal structures can make resection of large tumours even more difficult. The large space of the ischiorectal fossae facilitates asymptomatic growth [12], so these patients may present late, with large masses. Imaging—ideally MRI—can narrow the diagnostic considerations and evaluate extent and potential invasion of any surrounding structures to aid surgical planning [13].

This case is presented to increase the awareness of mammary-like glands of the anogenital region, which may give rise to fibroadenomas and like breast tissue, may harbour malignancy. Additionally, it raises awareness that fibroadenomas of the anogenital region can mimic the findings of soft tissue sarcoma—an important lesson for radiologists, general surgeons and gynaecologists.

## CONCLUSION

- Fibroadenoma arising from ectopic breast tissue is an important differential to consider for both gynaecologists and general surgeons reviewing perineal, vulva and perianal masses.
- Fibroadenomas of the anogenital region can mimic the findings of soft tissue sarcoma on imaging.
- Extra-mammary breast tissue has malignant potential and should be excised without delay.

## Data Availability

All data published in this report.
